# [Retracted] Regulation of miR-155 affects the invasion and migration of gastric carcinoma cells by modulating the STAT3 signaling pathway

**DOI:** 10.3892/ol.2025.15214

**Published:** 2025-08-04

**Authors:** Hua Wei, Yan Li, Qiang Ning, Zhi-Min Suo

Oncol Lett 16: 4137–4142, 2018; DOI: 10.3892/ol.2018.9152

Following the publication of the above paper, it was drawn to the Editor's attention by a concerned reader that the Transwell migration assay data shown in Fig. 1A and D on p. 4139 had already appeared in an article written by different authors at different research institutes that was published previously in the journal *Molecular Medicine Reports*.

Owing to the fact that the contentious data in the above article had already been published prior to its submission to *Oncology Letters*, the Editor has decided that this paper should be retracted from the Journal. The authors were asked for an explanation to account for these concerns, but the Editorial Office did not receive a reply. The Editor apologizes to the readership for any inconvenience caused.

